# A first report of *Streptococcus iniae* infection of the spotted sea bass (*Lateolabrax maculates*)

**DOI:** 10.3389/fvets.2024.1404054

**Published:** 2024-06-04

**Authors:** Yiqin Deng, Ziyang Lin, Liwen Xu, Jianjun Jiang, Changhong Cheng, Hongling Ma, Juan Feng

**Affiliations:** Key Laboratory of South China Sea Fishery Resources Exploitation and Utilization, Ministry of Agriculture and Rural Affairs, South China Sea Fisheries Research Institute, Chinese Academy of Fishery Sciences, Guangzhou, China

**Keywords:** *Streptococcus iniae*, spotted sea bass (*Lateolabrax maculates*), LD50, pathogenicity, antibiotic resistance

## Abstract

This study marks the first occasion that *Streptococcus iniae* has been isolated, identified, and characterized as the causative pathogen in spotted sea bass (*Lateolabrax maculates*). Infected fish exhibited a range of external symptoms, including scale loss, bleeding from the jaw, anus, and tail, among other signs, as well as internal manifestations such as congested liver, splenomegaly, branchial anemia, yellow fat syndrome, and intestinal edema. Notably, exophthalmia and meningoencephalitis—typical symptoms associated with previous *S. iniae* infections—were not observed. A predominant bacterial isolate (designated 10S01) was recovered from the pure culture of spleen of a diseased spotted sea bass in Zhuhai, China. The strain was then subjected to Gram staining, biochemical profiling, and molecular confirmation through 16S rRNA and *gyrB* gene, corroborating its identity as *S. iniae*. Pathogenicity was assessed by intraperitoneal injection challenge in spotted sea bass weighing approximately 13 g/fish, revealing a LD50 of 74 cfu/g-fish. The 10S01 strain demonstrated the ability to colonize various organs, including the spleen, liver, kidney, and brain, with a relatively higher affinity for the spleen. Furthermore, antimicrobial susceptibility testing indicated that the 10S01 strain was sensitive to 14 tested antibiotics, particularly chloramphenicol, ciprofloxacin, clarithromycin, florfenicol, ofloxacin, rifampicin, and trimethoprim/sulfamethoxazole, highlighting these as preferred treatments for *S. iniae* infections in spotted sea bass. These findings contribute significantly to our understanding of *S. iniae* pathogenesis and inform the prompt and appropriate antibiotic treatment of *S. iniae* infections.

## 1 Introduction

Aquaculture is an industry in robust expansion, flourishing to meet the consumer demand for high-quality food protein. In 2020, it produced a global output of 87.5 million tons, with China maintaining its position as the world's largest aquaculture producer. The yield of China constitutes a significant 35% of the total global production (1). Notably, fish farming contributes to 53% total aquaculture output of China. Among these, the spotted sea bass (*Lateolabrax maculatus*) stands out as an economically vital species, producing nearly 200,000 tons and accounting for ~11% of the marine cultured fish products in 2021 (2). Particularly in the Doumen district of Zhuhai, Guangdong Province, China, which represents the principal brackish water region for sea bass aquaculture in the country, boasts a culture area exceeding 26 million square meters. These areas are predominantly open-air semi-enclosed ecosystem ponds. The annual output from this region alone surpasses 50% total production of China (2), significantly contributing to the nation's animal protein supply and generating substantial economic benefits for local farmers.

As agricultural intensity and stocking densities escalate, aquaculture face increasingly greater risks from infectious diseases (3, 4). Our thorough investigation indicates that the most prevalent diseases affecting spotted sea bass stem from the infection of bacteria (*Aeromona, Edwardsiella, Vibrio*, and *Nocardia*), virus (*Iridovirus* and *Neuronecrosis virus*), and parasite (*Scuticociliatid ciliate, Trichodina, Dactylogyrus*, and *Ichthyophthirius*). With the expansion of aquaculture, host species may encounter novel pathogens, posing unexpected challenges and substantial burdens to the industry (5–7). A prime example is the emergence of Acute Hepatopancreatic Necrosis Syndrome (AHPNS) in shrimp, which was first detected in China in 2009 and subsequently spread to Vietnam (2010), Malaysia (2011), Thailand (2012), and Mexico (2013) (8). This led to a significant decline in global shrimp production from 2011 to 2013 (−5.7%: 2011–2012; and −9.6%, 2012–2013) (https://wenku.baidu.com/view/179efcb8da38376bae1fae77.html?fr=sogou&_wkts_=1681790963165). Until early 2013, the causative agent of AHPNS was identified as Vibrio parahaemolyticus (5). The identification of AHPNS pathogens and in-depth study of their pathogenic mechanisms have facilitated the rapid development of detection methods and effective disease prevention and control strategies. These efforts have contributed to the slow recovery of the global shrimp industry since 2014, as evidenced by the reduction in losses or increase in production in the primarily affected Asian countries (9). Therefore, the rapid and timely identification of new pathogens in cultured animals and the analysis of their pathogenicity and drug resistance are of great significance for the prevention and control of emerging diseases.

**Figure 1:**
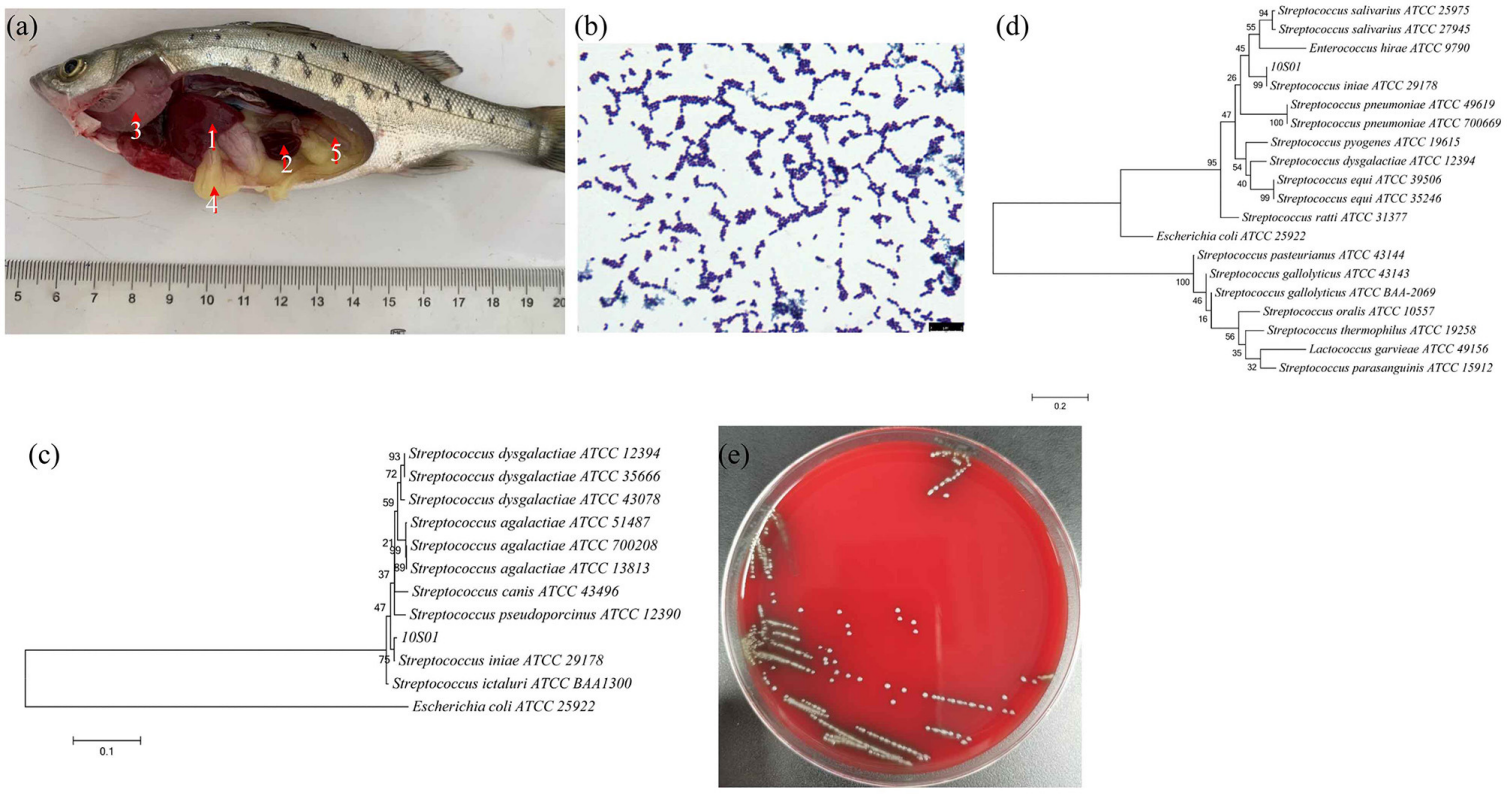
The visceral lesions of infected spotted sea bass (*Lateolabrax maculates*) upon dissection **(a)**, depicting the liver (1), spleen (2), gill (3), intestines (4), and kidney (5), and the gram staining **(b)**, the phylogenetic tree of 16S rRNA sequences **(c)**, the phylogenetic tree of *gyrB* sequences **(d)**, and the hemolysis **(e)** of the isolated strain 10S01. In the phylogenetic tree, the branch points are labeled with relative bootstrap values (in percentage, calculated by bootstrap values ÷ 1,000 × 100), and the scale bar represents 0.01 substitutions per sequence position.

Originally isolated from freshwater dolphins in 1976, *Streptococcus iniae* has emerged as one of the most serious aquatic pathogens over the past decade. It is the primary pathogen affecting a wide range of marine and freshwater fish species, including tilapia (*Oreochromis* spp.), rainbow trout (*Oncorhynchus mykiss*), red drum (*Sciaenops ocellatus*), and rabbit fish (*Siganus* spp.) (10). This bacterium was responsible for economic losses amounting to US $100 million worldwide in 1997 (10). In this study, we firstly identified *S. iniae* as the causative agent of spotted sea bass with characteristic symptoms such as congestive liver, splenomegaly, branchial anemia, yellowing fat, and edematous intestine (Figure 1a), resulting in nearly 10% mortality. A highly virulent strain of the bacteria was isolated from the infected spotted sea bass, and its pathological characteristics and drug resistance were analyzed. This information is instrumental for further research into its pathogenesis and for ensuring timely and effective antibiotic treatment.

## 2 Materials and methods

### 2.1 Fish sampling and bacterial isolation

Diseased spotted sea bass (*L. maculatus*) were collected from a brackish water fish farm located in Zhuhai, Guangdong Province, China. Fish exhibiting moribund condition and characteristic clinical symptoms such as congestive liver, splenomegaly, branchial anemia, yellowing fat, and edematous intestine were selected for bacteriological examination. Prior to dissection, the skin of the fish was thoroughly cleaned with 75% ethanol. Sterile blades were used to excise samples of the spleen, liver, heart, kidney, and gallbladder using sterile blades. The inoculation loop was then used to touch the incision and streak the tissue fluid onto Brain Heart Infusion (BHI) agar plates for bacterial isolation. The plates were incubated at 28°C for 24 hours, and dominant uniform bacterial colonies were isolated by restreaking onto BHI agar plates twice. A single bacterial colony was picked and cultured in BHI broth at 28°C for 16 h before being stored at −80°C in BHI broth supplemented with 15% (v/v) sterile glycerol. A predominant strain was provisionally designated as 10S01.

### 2.2 Gram staining, hemolysis, and biochemical characterization

Gram staining was performed using the Stain Kit 029010 (Huankai Microbial, Guangzhou, China) following the manufacturer's protocol. The stained bacterial cells were examined using an optical microscope (Leica, Germany). Bacterial hemolysis was assessed by streaking the 10S01 strain onto a 5% sterile defibrinated sheep blood agar plate. A total of 43 biochemical tests were conducted using the Vitek 2 Compact 60 (Biomerieux, France) GP card, adhering to the manufacturer's guidelines. The identification of the bacterial species was determined based on the pattern of biochemical reactions observed.

### 2.3 16S rRNA and *gyrB* gene amplification and sequencing

The genomic DNA of strain 10S01 was extracted using the Bacterial Genomic DNA Extraction Kit DP302 (Tiangen-Biotech, Beijing, China) following the manufacturer's protocol and stored at −20°C until further use. For the amplification of the 16S rRNA gene, the universal primer set 8 F (AGAGTTTGATCCTGGCTCAG) and 1492 R (GGTTACCTTGTTACGACTT) was employed. The specific primer pair F3 (GAAGATGATTCCATTACCGTTG) and R3 (CTAAATCTTCTCCTACCACACC) was used for the amplification of the *gyrB* gene (11). The amplified products were sequenced, and a BLAST search was conducted via the NCBI website (https://www.ncbi.nlm.nih.gov/). Subsequently, the top 1,000 homologous ATCC strains were selected for the construction of phylogenetic trees using the neighbor-joining method in MEGA6.0 software (12). *Escherichia coli* ATCC 25922 (13) was chosen as the outgroup strain for reference.

### 2.4 spotted sea bass challenge assay

A challenge trial was conducted to ascertain the median lethal dose (LD50) of strain 10S01 in spotted sea bass. The fish, weighing an average of 12.82 g, were kept in aerated ponds with water temperatures maintained at 28±1°C. They received a basal diet equivalent to 5% of their body weight twice daily (at 7 am and 6 pm) and were fasted for 2 days prior to infection. A pure culture of 10S01 was streaked onto a BHI agar slope and incubated overnight at 28°C. Subsequently, the bacterial cells were resuspended in saline and adjusted to concentrations of 3 × 10^1^, 3 × 10^2^, 3 × 10^3^, 3 × 10^4^, and 3 × 10^5^ cfu/mL, respectively. Intraperitoneal injections were administered using 100 μL of the bacterial suspension at each concentration. One hundred and twenty-six spotted sea bass were randomly selected, anesthetized with eugenol (the volume ratio of eugenol/water = 1/100,000), and intraperitoneally injected with 100 μL of the bacterial suspension at the various concentrations. Each concentration was tested on 21 fish, which were evenly distributed into three 100 L plastic buckets. The control group received an intraperitoneal injection of 100 μL of 0.85% saline solution and was divided equally into three 100 L aerated plastic buckets, with 7 fish per bucket. Mortality was recorded over a period of 7 days. The LD50 was calculated using the method described by Sun Ruiyuan Käber, applying the formula lgLD50 = XK - i(∑p – 0.5), where “XK” is the logarithm of the maximum dose, “i” is the logarithm of the ratio between two adjacent doses, and ∑p represents the cumulative mortality across all groups.

### 2.5 Sampling, pathogen re-isolation and identification, and histopathological analysis

Three fish exhibiting characteristic symptoms such as splenomegaly, liver congestion, scale loss, and/or bleeding from the jaw, anus, and/or tail were selected for dissection to facilitate pathogen re-isolation and histopathological examination. For pathogen re-isolation, the liver, spleen, and kidney were excised using a sterile surgical blade. The tissue homogenate was then collected with an inoculation loop and streaked onto BHI agar plates. The predominant clone was purified twice on BHI plates and identified through PCR amplification and sequencing of the 16S rRNA gene. For histopathological analysis, tissues including the brain, heart, liver, spleen, gills, intestines, and stomach from each fish were fixed in 10% buffered formalin for a minimum of 24 h. Three fish from the control group were dissected for comparison. The tissues were dehydrated, embedded in paraffin, and sectioned at 4 micrometers using a rotary microtome (RM2135, Leica, Wetzlar, Germany). The sections were stained with hematoxylin and eosin (HE) using standard techniques for histopathological assessment. All slides were examined under an optical microscope (Leica DFC495, Ernst Leitz, Wetzlar, Germany).

### 2.6 Colonization of 10S01 in fish

Fifty fish were selected for injection with 100 μL of a 3 × 10^5^ cfu/mL concentration of the 10S01 strain. At 0, 12, 24, 36, 48, and 60 h post-injection, three fish were dissected. Approximately 100 mg of brain, liver, spleen, and kidney tissue from each fish were excised and immersed in 100% ethanol for DNA extraction and subsequent bacterial load quantification using absolute quantitative PCR (qPCR).

Total DNA was extracted from the tissues following the protocol of the Marine Animal Tissue Genomic DNA Extraction Kit (TIANGEN, DP324). The *gyrB* gene was targeted for the absolute quantification of the bacterial load of 10S01. Initially, the *gyrB* gene was amplified and cloned into a pUC19 vector. The recombinant vector was then extracted, and its concentration was determined using a NanoDrop 2000 spectrophotometer. The copy number of the recombinant vector was calculated based on the vector concentration and molar mass. The recombinant vector was serially diluted, and qPCR was performed using the diluted vectors as templates. A standard curve was constructed using the logarithm of the template DNA copy number as the x-axis and the cycle threshold (CT) values as the y-axis. Subsequently, qPCR was carried out with the tissue DNA as templates, and the bacterial copy numbers were determined by correlating the CT values with the standard curve.

### 2.7 Antibiotics susceptibility test

Antibiotics susceptibility of the strain 10S01 was determined by a disk diffusion method on Mueller–Hinton agar using commercial antibiotic discs (Oxoid) according to Clinical and Laboratory Standards Institute (CLSI) guidelines (14). The *Escherichia coli* ATCC 35218 strain served as the quality control organism. The panel of 17 antibiotics tested encompassed ampicillin (10 μg/disc), amoxicillin (10 μg/disc), cephalexin (300 μg/disc), cefazolin (30 μg/disc), tetracycline (30 μg/disc), doxycycline (30 μg/disc), erythromycin (15 μg/disc), clarithromycin (15 μg/disc), ciprofloxacin (5 μg/disc), ofloxacin (5 μg/disc), sulfaisoxazole (300 μg/disc), trimethoprim/sulfamethoxazole (1.25/23.75 μg/disc), chloramphenicol (300 μg/disc), florfenicol (10 μg/disc), kanamycin (30 μg/disc), rifampicin (5 μg/disc), and streptomycin (300 μg/disc). The interpretation of inhibition zones was conducted in accordance with the manufacturers' guidelines and the Clinical and Laboratory Standards Institute (CLSI) M45-A document (15).

## 3 Results

### 3.1 Isolation and identification of *S. iniae* 10S01 strain

The strain SB210310S01, referred to as 10S01, was successfully isolated from the spleen tissue of a diseased sea bass, as depicted in Figure 1a. Microscopic examination revealed the bacteria stained in purple and arranged characteristically in chains, as seen in Figure 1b, suggesting its classification as a typical Gram-positive member of the Streptococcus genus. Biochemical profiling using the Vitek 2 Compact 60 GP card revealed a pattern of metabolic activities for *S. iniae* 10S01, with 17 biochemical reactions returning positive results and 22 negative (Table 1). Four reactions were ambiguous due to inconsistencies across triplicate testing (Table 1). The strain demonstrated the capacity to utilize dRIB, NAG, dMAL, dMAN, dMNE, SAL, SAC, and dTRE as carbon source and to produce enzymes such as APPA, LeuA, BGURr, PyrA, BGUR, AlaA, TyrA, and dRIB. Additionally, resistance was observed against POLYB and OPTO. Conversely, the strain could not metabolize AMY, dXYL, CDEX, dSOR, lLATk, LAC, MBdG, PUL, and dRAF, nor could it produce the enzymes PIPLC, ADH1, BGAL, AspA, BGAR, AMAN, PHOS, AGAL, and URE. Sensitivity to PUL and O129R was noted, along with an inability to grow in the presence of 6.5% NaCl. Biochemical assays indicated a probability >88% that the isolate belonged to the *Streptococcus* genus. Further corroboration was achieved through the construction of phylogenetic trees based on 16S rRNA and *gyrB* gene sequences. These analyses clustered strain 10S01 closely with the type strain *S. iniae* ATCC 29178, clearly differentiating it from other *Streptococcus* species strains, as shown in Figures 1c, d. Integrating Gram staining, biochemical tests, and phylogenetic analysis confirmed the classification of strain 10S01 as a member of *S. iniae*. Finally, alpha (incomplete) hemolysis was observed on blood agar plates containing 5% sterile defibrinated sheep blood, as illustrated in Figure 1e.

**Table 1:** The physiological and piochemical analysis of *S. iniae* 10S01.

**Reaction**	**Result**	**Reaction**	**Result**	**Reaction**	**Result**	**Reaction**	**Result**	**Reaction**	**Result**	**Reaction**	**Result**
AMY	–	PIPLC	–	dXYL	–	ADH1	–	BGAL	–	AGLU	/
APPA	+	CDEX	–	AspA	–	BGAR	–	AMAN	–	PHOS	–
LeuA	+	ProA	/	BGURr	+	AGAL	–	PyrA	+	BGUR	+
AlaA	+	TyrA	+	dSOR	–	URE	–	POLYB	+	dGAL	/
dRIB	+	lLATk	–	LAC	–	NAG	+	dMAL	+	BACI	–
NOVO	/	NC6.5	–	dMAN	+	dMNE	+	MBdG	–	PUL	–
dRAF	–	O129R	–	SAL	+	SAC	+	dTRE	+	ADH2s	–
OPTO	+										

+, capacity to utilize the carbon source, can produce the enzymes, or resistant to the antibiotic; –, cannot utilize the carbon source, cannot produce the enzymes, or sensitivity to the antibiotic; /, the reactions are ambiguous. AMY, Amygdalin; PIPLC, Phosphatidylphospholipase C; dXYL, D-xylose; ADH 1, Arginine dihydrolase 1; BGAl, Beta-D-galactosidase; AGLU, alpha-glucosidase; APPA, Alanine-phenylalanine-proline arylaminase; CDEX, Cyclodextrin; AspA, L-aspartate arylaminase; BGAR, beta-galactosyranosidase; AMAN, alpha-mannosidase; PHOS, Phosphatase; LeuA, Leucine arylaminase; ProA, L-proline arylaminase; BGURr, Beta-glucuronidase; AGAL, alpha-galactosidase; PyrA, Pyroglutamate arylaminase; BGUR, beta-D-glucuronidase; AlaA, Alanine arylaminase; TyrA, Tyrosine arylaminase; dSOR, D-sorbitol; URE, urease; POLYB, Polymyxin B tolerance; dGAL, D-galactose; dRIB, d-ribose; lLATk, L-lactate produces alkali; LAC, lactose; NAG, N-acetyl-D-glucosamine; dMAL, D-maltose; BACI, Bacitracin tolerance; NOVO, Neomycin tolerance; NC6.5, 6.5%NaCl growth; dMAN, D-mannitol; dMNE, D-mannose; MBdG, Methyl-B-D-glucopyranoside; PUL, Amylopectin; dRAF, D-raffinose; O129R, O/129 tolerance; SAL, Salicin; SAC, saccharose; dTRE, D-trehalose; ADH2s, Arginine dihydrolase 2; OPTO, Optokhin tolerance.

### 3.2 Virulence and histopathologic characterization of *S. iniae* 10S01 in spotted sea bass

**Figure 2:**
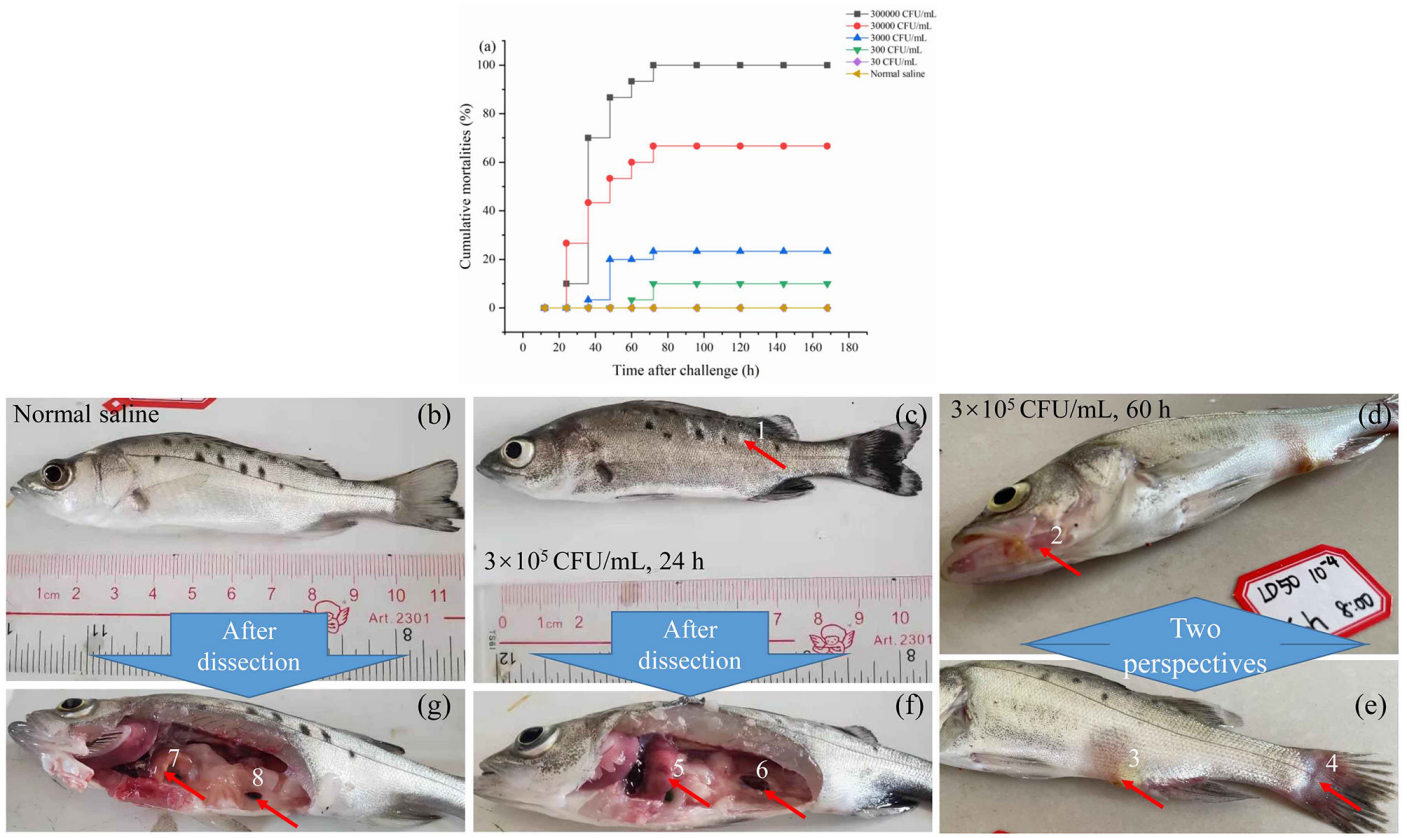
The mortality rate within 7 days after injection of *S. iniae* 10S01 at varying concentrations **(a)**, pathological features of spotted sea bass infected with normal saline **(b, g)**, *S. iniae* 10S01 at a concentration of 3.0 × 10^5^ cfu/mL for 24 h **(c, f)** and 60 h **(d, e)** (Pathological changes include: 1, scale loss; 2–4, bleeding from the jaw, anus, and tail, respectively; 5, liver congestion; 6 splenomegaly; 7, normal liver; 8, normal spleen).

As illustrated in Figure 2a, following injection, fish mortalities were recorded at 24 h for the concentrations of 3.0 × 10^5^ and 3.0 × 10^4^ cfu/mL. However, no deaths occurred beyond 72 h post-injection at any tested concentration. The cumulative mortality rates were 10%, 70%, 86.7%, 90.3%, and 100% at 24, 36, 48, 60, and 72 h post-infection, respectively, with an injection concentration of 3.0 × 10^5^ cfu/mL. With the concentration of 3.0 × 10^1^ cfu/mL, no fish died during the observation period up to 144 h. The lethal dose 50% (LD50) was determined to be 74 cfu/g of fish using the modified Sun Ruiyuan Käber Method.

**Figure 3:**
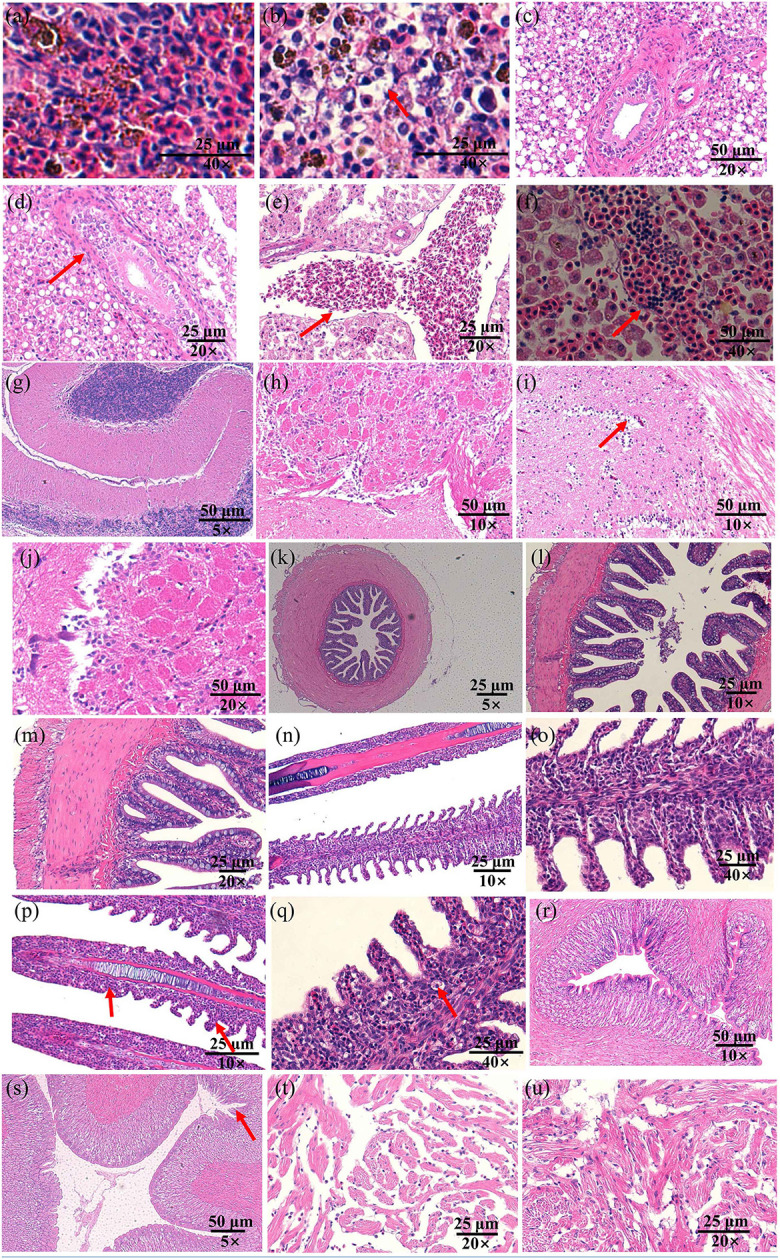
The histopathological changes in the spleen **(a, b)**, liver **(c–f)**, brain **(g–j)**, gut **(k–m)**, gill **(n–q)**, stomach **(r, s)**, and heart **(t, u)** of spotted sea bass following infection with *S. iniae* strain 10S01. Panels **(a, c, e, g, h, k, n, o, r, t)**, show tissues treated with normal saline, while **(b, d, f, i, j, l, m, p, q, s, u)** illustrate tissues from fish infected with *S. iniae* 10S01. Arrows indicate the presence of lesions.

**Figure 4:**
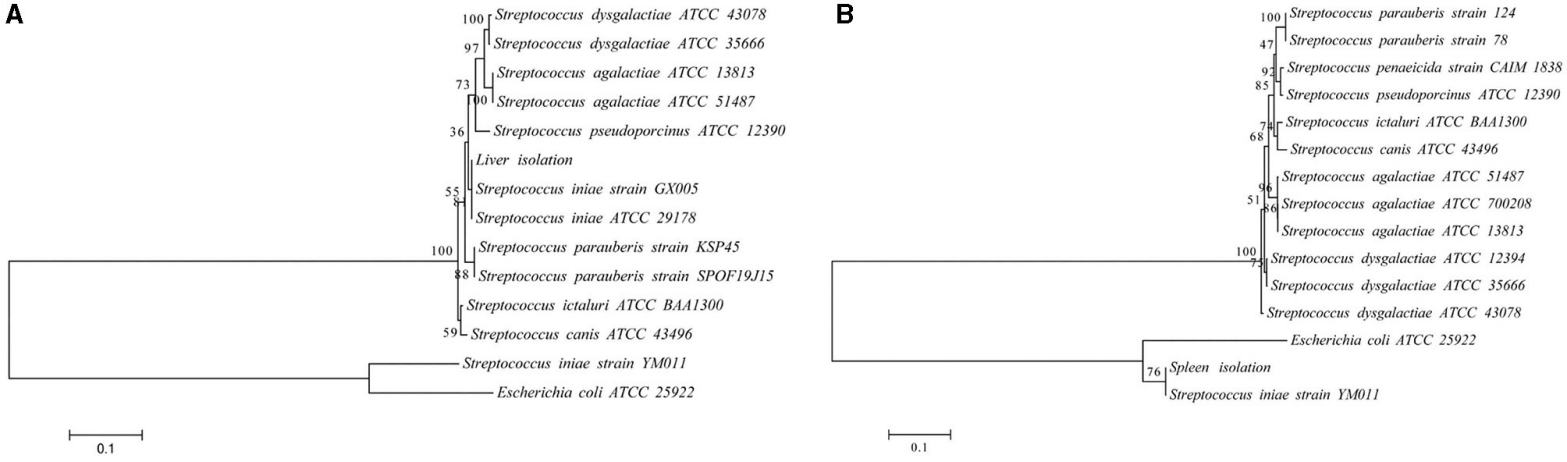
The phylogenetic trees of the 16S rRNA **(A)** and *gyrB* **(B)** sequence with representative microbes aligned by using MEGA 6.0. The branch points are labeled with the relative bootstrap values (expressed as a percentage, calculated by bootstrap values ÷ 1,000 × 100). The scale bar represents 0.01 substitutions per sequence position.

Infected fish exhibited signs of lethargy, anorexia, twitching, scale loss, and occasional bleeding from the jaw, anus, and tail, as shown in Figures 2b–e. Upon dissection, splenomegaly and liver congestion were evident (Figures 2f, g). Histopathological analysis results are presented in Figure 3. Normal saline group characteristics are seen in Figure 3a (spleen), c (liver), g and h (brain), k (intestine), n and o (gill), r (stomach), and t (heart). In comparison to the control group, the spleen displayed severe parenchymal cell damage, cortical cavitation, and fiber disruption (Figure 3b). Hepatic tubule epithelium desquamation, hepatic blood cell infiltration, liver cell atrophy, and lymphocyte accumulation were observed (Figures 3d–f). Brain cell vacuolation was noted (Figure 3i). The gill showed hyperplasia in gill lamellae and lamellar fusion (Figure 3p). And partial cells of the gill filament showed cavitation (Figure 3q). The left gastric mucosa was swollen while the right side was atrophied (Figure 3s). No significant pathology was detected in the intestine and heart (Figures 3l, m, u). Both dominant clones re-isolated from the enlarged spleen and congested liver were identified as *S. iniae* (Figures 4A, B).

### 3.3 Colonization dynamics of *S. iniae* 10S01 in spotted sea bass

**Figure 5:**
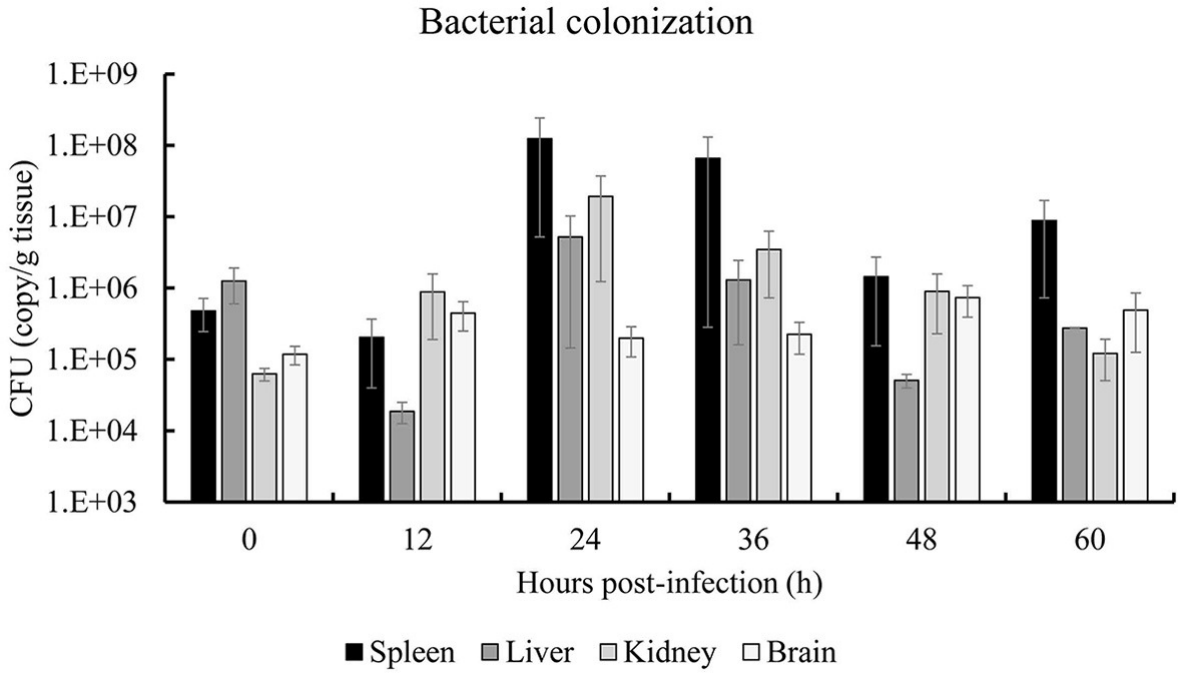
Bacterial colonization of spleen, liver, kidney and brain of spotted sea bass (*Lateolabrax maculates*) by *S. iniae* 10S01. Values are means ± SEM (*n* = 3).

The colonization dynamics of *S. iniae* 10S01 within the spotted sea bass are illustrated in Figure 5. Pathogen load analysis revealed that strain 10S01 exhibited a relatively higher colonization rate in the spleen compared to other organs. The pathogen load in both the spleen and liver exhibited a similar trend over time. Initially, the colonization load decreased from 0 to 12 h post-injection (hpi), then increased, peaking at 24 hpi, followed by a decline until 48 hpi, and finally rose again at 60 hpi. In the kidney, the pathogen load ascended from 0 to 24 hpi before declining through 60 hpi. Conversely, in the brain, the pathogen load remained relatively constant throughout the extended injection period.

### 3.4 Antibiotic susceptibility profiling

Strain 10S01 demonstrated susceptibility to 14 different antibiotics tested, including ampicillin, amoxicillin, cephalexin, cefazolin, tetracycline, doxycycline, erythromycin, clarithromycin, ciprofloxacin, ofloxacin, trimethoprim/sulfamethoxazole, chloramphenicol, florfenicol, and rifampicin. It exhibited intermediate susceptibility to kanamycin and streptomycin, while resistance was observed against sulfaisoxazole (Table 2).

**Table 2:** The antibiotics resistance of *S. iniae* 10S01.

**Antibiotics**	**Concentration (μg/ per disk)**	**Size of inhibition zone (mm)**	**R/I/S**
Ampicillin	10	50 ± 2.1	S
Amoxicillin	10	55 ± 1.8	S
Cephalexin	300	40 ± 1.6	S
Cefazolin	30	55 ± 1.3	S
Chloramphenicol	300	40 ± 2.9	S
Ciprofloxacin	5	31 ± 2.1	S
Clarithromycin	15	40 ± 2.1	S
Doxycycline	30	36 ± 1.2	S
Erythromycin	15	36 ± 1.5	S
Florfenicol	30	38 ± 2.5	S
Kanamycin	30	15 ± 1.2	I
Ofloxacin	5	28 ± 1.5	S
Rifampicin	5	35 ± 1.5	S
Streptomycin	300	15 ± 1.4	I
Sulfaisoxazole	300	10 ± 1.7	R
Trimethoprim/sulfamethoxazole	1.25/23.75	25 ± 1.1	S
Tetracycline	30	38 ± 1.9	S

R, resistant; I, intermediate; S, susceptible.

## 4 Discussion

*S. iniae* is a causative agent of streptococcicosis in various wild and farmed fish species of economic importance, ranking as one of the principal pathogens responsible for high mortality rates and significant economic losses (16). To date, *S. iniae* has been reported to infect over 30 different fish species, such as striped bass, tilapia, rainbow trout, red drum, red porgy, rabbitfish, olive flounder, barramundi, and Chinese sturgeon (17). However, there have been no previous reports of infection in the spotted sea bass. In our current study, we discovered for the first time that *S. iniae* can indeed infect spotted sea bass resulting in approximately 10% mortality. The infection happened in Zhuhai, China, a major cultivation area for this species.

Infections caused by *S. iniae* can manifest as acute or subacute forms, with the primary symptom being meningoencephalitis in fish. Acutely infected fish often succumb rapidly, with a mortality rate exceeding 50%, yet without displaying characteristic symptoms (18). In contrast, subacute infections result in a lower mortality rate, though daily deaths occur during the infection period. Additionally, fish infected with *S. iniae* display a variety of pathological symptoms (19). For instance, the infection in tilapia (Oreochromis aureus) typically involves conditions such as meningitis, epi/myocarditis, and septicemia, accompanied by splenomegaly or necrosis in the spleen and kidneys. Moreover, infected tilapia often exhibit loss of appetite, imbalance and erratic swimming behaviors, a tendency to swim near the water's surface, scale detachment, hemorrhages on various parts of the body surface, mild abdominal dropsy, mild exophthalmia (either unilateral or bilateral), along with skin erosions and ulcerations (20). Furthermore, *S. iniae* is also implicated in causing hemorrhagic septicemia in olive flounders (*Paralichthys olivaceus*) (21). In this study, we identified a subacute infection of *S. iniae* in spotted sea bass, presenting with typical symptoms such as hepatic congestion, splenomegaly, branchial pallor, yellowing of fat, and intestinal edema (Figure 1a), resulting in nearly 10% mortality. However, the typical symptom of exophthalmos associated with *S. iniae* infection was not observed. A potential pathogenic strain was isolated from the spleen, designated as strain 10S01. Further analysis of its pathological characteristics and antibiotic resistance profiles is instrumental for elucidating its pathogenesis and facilitating timely antibiotic treatment.

Bacterial identification utilizing biochemical, moleculartypic, and phenotypic analysis methods has become a standard practice, complemented by morphological analysis. Gram staining is a widely adopted differential technique in bacteriology, offering a clear contrast between bacteria and their surroundings, thereby allowing for the observation of bacterial morphology, arrangement, and other structural features (22). In this study, strain 10S01 was observed to be stained purple and arranged in chains, characteristics indicative of a typical Gram-positive Streptococcus species. The VITEK^®^ 2 Compact system, grounded in extensive manual verification experience, offers an automated identification solution (23). With barcode-identified operations, it ensures complete traceability and minimizes human error. Additionally, leveraging advanced colorimetric technology, VITEK^®^ 2 Compact simultaneously analyzes multiple reactions to enhance result accuracy. Based on Gram staining outcomes, the GP card was employed in this investigation. Due to the limitations of the system's database, strain 10S01 was identified as a Streptococcus species. It was found to metabolize eight carbon sources, including dMNE, and dTRE, and produce eight enzymes, such as LeuA. These biological traits mirror those of S. iniae strains previously isolated from cultured flounder in Jeju (24). Additionally, strain 10S01 demonstrates a tolerance to POLYB and OPTO. Notably, strain 10S01 exhibits sensitivity to BAC, contrasting with the flounder-infecting *S. iniae* strain 1083 which shows resistance to BAC, as reported by Lee et al. (24). Furthermore, strain 10S01 is incapable of utilizing LAC as a carbon source, whereas the majority of flounder-infecting *S. iniae* strains are capable of metabolizing LAC (24). The 16S rRNA gene serves as a universal marker for bacterial identification across all bacterial species (25). However, due to homology among bacteria, 16S rRNA often fails to provide species-level identification, necessitating the use of species-specific genes like *gyrB* for further assistance. Phylogenetic trees based on both 16S rRNA and *gyrB* sequences indicated that strain 10S01 clustered with *S. iniae*. Additionally, *S. iniae* has the ability to display both alpha and beta hemolysis. For instance, beta hemolysis was observed in *S. iniae* isolates from the Japanese flounder (*Paralichthys olivaceus*) as reported by Matsuoka et al. (26), and in the golden pompano (*Trachinotus ovatus*) from China, as documented by Cai et al. (27). In contrast, the strain 10S01 exhibits alpha hemolytic activity. Collectively, strain 10S01 has been conclusively identified as *S. iniae*.

Following bacterial identification, it is imperative to confirm whether the isolated strain is indeed the primary causative agent of the infection. In accordance with Koch's postulates, a deliberate infection test is essential to establish the pathogenicity of the strain in question. In this instance, the pathogenicity of strain 10S01 was assessed, revealing a relatively low lethal dose (LD50) for Spotted sea bass at 74 cfu/g-fish. This value surpasses that reported for the S. iniae strain initially isolated from red hybrid tilapia (1.5 cfu/g-fish) (28), yet it is lower than those recorded for red porgy (*Pagrus pagrus*, L.) (378 cfu/g-fish) (29) and Chinese sturgeon (*Acipenser sinensis*) (1.6 x 10^4^ CFU/g-fish) (30). Similar to natural infections, the experimentally infected fish exhibited splenomegaly and hepatic congestion. Histopathological analysis further revealed tissue lesions in the spleen, liver, brain, gills, and stomach. Notably, acute meningitis—characterized by inflammatory cell infiltration in the meningeal blood vessels and the presence of granuloma—was not observed, a feature that has been seen in red porgy (*Pagrus pagru*s, L.) (29) and tilapia (*O. aureus*) (31). Colonization assays corroborated that *S. iniae* can indeed invade the spleen, liver, and brain of spotted sea bass, demonstrating a predilection for splenic colonization. Consequently, it is evident that *S. iniae* represents the principal pathogen responsible for this outbreak, and strain 10S01 may serve as a model for investigating its pathogenic mechanisms in spotted sea bass, as well as potentially serving as a basis for developing a live attenuated vaccine.

Compared to vaccination, antibiotic therapy offers a more immediate and efficacious treatment option for emerging diseases. However, the overuse of antibiotics can lead to the emergence of resistance, posing threats to both the environment and food safety. Consequently, the use of aquatic antibiotics is subject to stringent global regulations. In China, for instance, aquaculture is permitted to utilize 11 specific antibiotics, including sulfoxamycin, flufenicol, flumequine, enrofloxacin, doxycycline hydrochloride, ciprofloxacin, neomycin sulfate, sulfamethoxine sodium, compound sulfadiazine, compound sulfadipyrimidine, and compound sulfamethoxazole (32). The U.S. Food and Drug Administration (FDA) has approved four drugs—florfenicol, oxytetracycline dihydrate, sulfadimethoxine & ormetoprim, and sulfamerazine—as Type A medicated articles for the production of medicated feed intended for food fish (source: https://www.fda.gov/animal-veterinary/product-safety-information/letter-aquaculture-professionals). In Japan, while over 20 antibacterial agents are approved for aquaculture, their use is highly specific, being designated for particular fish species and diseases (33). For example, sulfadimethoxine is allowed for use exclusively in the treatment of rainbow trout disease (34). Furthermore, analyzing antibiotic resistance is crucial for the success of chemotherapeutic treatments, thereby reducing mortality rates and lessening the impact on production. For instance, Ortega et al. (31) documented the initial infection of *S. iniae* in tilapia farmed in Querétaro, Mexico. The isolated pathogenic *S. iniae* strain was found to be sensitive to oxytetracycline, and records from the facility indicated that the infected fish were treated with this antibiotic, leading to a significant decrease in daily mortalities. (31). In our study, we discovered that the isolated strain 10S01 exhibited sensitivity to eleven tested antibiotics, a finding that aligns with the antibiotic sensitivity profiles of *S. iniae* strains from infected tilapia, red porgy (*Pagrus pagrus* L), and Chinese Sturgeon (*Acipenser sinensis*) (29–31, 35). Specifically, all isolates demonstrated a high degree of sensitivity to chloramphenicol, ciprofloxacin, clarithromycin, florfenicol, ofloxacin, rifampicin, and trimethoprim/sulfamethoxazole. In accordance with the antibiotics approved for use within our country, ciprofloxacin and florfenicol are recommended as the primary therapeutic agents for treating *S. iniae* infections in spotted sea bass.

## 5 Conclusion

In our investigation, we documented the initial occurrence of *S. iniae* infection in spotted sea bass, which resulted in lesions to the liver, spleen, gills, and intestines. Notably, this strain did not elicit the characteristic symptoms of exophthalmos and meningoencephalitis. The isolation of the highly virulent strain 10S01 paves the way for further research into its pathogenesis and the development of vaccines. Antibiotic susceptibility testing revealed that chloramphenicol, ciprofloxacin, clarithromycin, florfenicol, ofloxacin, rifampicin, and trimethoprim/sulfamethoxazole are effective against *S. iniae* infections in spotted sea bass. Moving forward, we advocate a three-pronged approach: (1) employing an interdisciplinary strategy to unravel the Pathogenesis of *S. iniae* strain 10S01, which will aid in the creation of attenuated live vaccines and genetically engineered vaccines; (2) isolating more pathogenic strains and selecting representative strains based on molecular typing to develop inactivated vaccines; (3) screening for probiotics, plants, and bacteriophages that can inhibit *S. iniae* infection to develop biological control strategies.

## Data availability statement

The original contributions presented in the study are included in the article/supplementary material, further inquiries can be directed to the corresponding author.

## Ethics statement

The animal study was approved by the experimental procedures in the current study were carried out in strict compliance with the pertinent guidelines and regulations set forth by the Committee on Laboratory Animal Welfare and Ethics of South China Sea Fisheries Research Institute (nhdf2023-08). The study was conducted in accordance with the local legislation and institutional requirements.

## Author contributions

YD: Conceptualization, Data curation, Formal analysis, Funding acquisition, Investigation, Methodology, Writing—original draft, Writing—review & editing. ZL: Data curation, Formal Analysis, Investigation, Software, Writing—review & editing. LX: Formal Analysis, Investigation, Methodology, Writing—review & editing. JJ: Investigation, Methodology, Writing—review & editing. CC: Investigation, Software, Writing—original draft. HM: Formal analysis, Investigation, Writing—original draft. JF: Conceptualization, Funding acquisition, Project administration, Supervision, Writing—review & editing.
